# 1095. Increased Risk of COVID-19 Hospitalization and Death in Vaccinated Patients with End-Stage Renal Disease (ESRD) and Dialysis: Initial Results from INFORM, a Retrospective Health Database Observational Study in England

**DOI:** 10.1093/ofid/ofad500.068

**Published:** 2023-11-27

**Authors:** Sabada Dube, Yi Lu, Richard McNulty, Sophie Graham, Sofie Arnetorp, Nahila Justo, Renata Yokota, Kathryn Evans, Sudhir Venkatesan, Mark Yates, Sylvia Taylor, Jennifer Quint, Rachael A Evans

**Affiliations:** Medical Evidence, Vaccines and Immune Therapies Unit, AstraZeneca, Cambridge, UK;, Cambridge, England, United Kingdom; Real-World Evidence, Data Analytics, Evidera, London, UK, London, England, United Kingdom; Medical Affairs, Vaccines and Immune Therapies Unit, AstraZeneca, Cambridge, UK, Cambridge, England, United Kingdom; Real-World Evidence, Data Analytics, Evidera, London, UK, London, England, United Kingdom; Health Economics and Payer Evidence, BioPharmaceuticals R&D, AstraZeneca, Gothenburg, Sweden, Gothenburg, Vastra Gotaland, Sweden; Real-World Evidence, Data Analytics, Evidera, Stockholm, Sweden and Department of Neurobiology, Care Science and Society, Karolinska Institute, Stockholm, Sweden, Stockholm, Sodermanlands Lan, Sweden; P95, Leuven, Belgium, Dilbeek, Luxembourg, Belgium; Real-World Evidence, Data Analytics, Evidera, Waltham, MA, USA, Waltham, Massachusetts; Medical and Payer Evidence Statistics, BioPharmaceutical Medical, AstraZeneca, Cambridge, UK, Cambridge, England, United Kingdom; Real-World Evidence, Data Analytics, Evidera, London, UK, London, England, United Kingdom; Medical Evidence, Vaccines and Immune Therapies Unit, AstraZeneca, Cambridge, UK, Cambridge, England, United Kingdom; National Heart and Lung Institute, Imperial College London, London, UK, Cambridge, England, United Kingdom; University of Leicester, Leicester, England, United Kingdom

## Abstract

**Background:**

Individuals with immunocompromised conditions (IC) have a higher risk of COVID-19 morbidity and death despite vaccination. Though patients (pts) with ESRD are considered immunocompromised and have a weaker antibody response to COVID-19 vaccination, some guidelines do not currently recommend pre-exposure prophylaxis (PrEP) for these pts. This is due to the fact that boosters are believed to increase seropositivity and antibody production in pts on dialysis. The INFORM study aimed to describe the health burden of severe COVID-19, and here initial results are presented for fully vaccinated pts with ESRD or dialysis (ESRD or D); fully vaccinated pts with ESRD receiving dialysis (ESRD+D), and the fully vaccinated overall population (OP) aged ≥ 12 years or a subgroup aged ≥ 65 years.

**Methods:**

The proportions, rates per 100 person-years (PY), and risks of COVID-19–related outcomes (hospitalizations, intensive care unit admissions, and mortality) in fully vaccinated (≥ 3 doses) pts aged ≥ 12 years in England, UK were identified using the National Health Service (NHS) databases between Jan 1, 2022–Dec 31, 2022. Only outcomes with COVID-19 recorded as the primary diagnosis were included.

**Results:**

A total of 19,450 (0.3%) pts with ESRD or D, including 8390 (0.1%) with ESRD+D, were identified from the OP of 7,180,205. The proportion of pts with ≥ 1 hospitalization was higher in pts with ESRD or D (2.4%) and with ESRD+D (3.1%) compared with the OP aged ≥ 12 years (0.2%) and pts aged ≥ 65 years (0.6%). Similarly, mortality for both pts with ESRD or D and those with ESRD+D was estimated at 0.5%; higher than the OP (0.05%). Incidence rates per 100 PY for hospitalization and COVID-19 deaths were ≥ 10× higher in both groups versus the OP aged ≥ 12 years, and ≥ 3.5× higher versus pts aged ≥ 65 years (**Table 1**).

**Figure:**
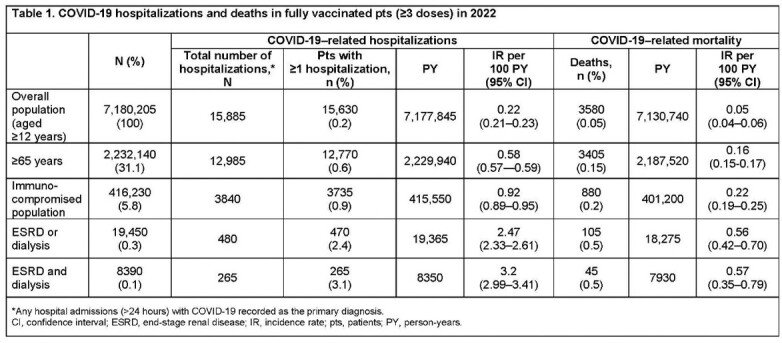

**Conclusion:**

Pts with IC mount inadequate responses to COVID-19 vaccines. In this study, pts with ESRD and/or receiving dialysis were also at an increased risk with higher rates of severe COVID-19 outcomes compared with the OP, despite vaccination. This risk is particularly elevated for pts with ESRD who are receiving dialysis. As a result, like pts with IC with an inadequate response to vaccines, pts with ESRD or D and ESRD with D may benefit from PrEP in addition to protection from vaccines against severe COVID-19.

**Disclosures:**

**Sabada Dube, PhD**, AstraZeneca: Employee **Yi Lu, PhD**, Evidera: Employee **Richard McNulty, MD**, AstraZeneca: Employee **Sophie Graham, MSc**, Evidera: Employee **Sofie Arnetorp, MS**, AstraZeneca: Employee **Nahila Justo, PhD, MBA**, Evidera: Employee|Karolinska Institute: Employee **Renata Yokota, PhD**, AstraZeneca: Employee **Kathryn Evans, MPH**, Evidera: Employee **Sudhir Venkatesan, MPH, PhD**, AstraZeneca: Employee **Mark Yates, PhD**, Evidera: Employee **Sylvia Taylor, PhD, MPH, MBA**, AstraZeneca: Stocks/Bonds **Jennifer Quint, PhD**, AstraZeneca: Grant/Research Support|Evidera: Grant/Research Support|GlaxoSmithKline: Grant/Research Support|Insmed: Grant/Research Support **Rachael A. Evans, PhD FRCP**, AstraZeneca: Advisor/Consultant|Boehringer: Advisor/Consultant|Evidera: Advisor/Consultant

